# The National Council of Women

**Published:** 1920-10-23

**Authors:** 


### THE NATIONAL COUNCIL OF WOMEN.

NOTES BY A SOCIAL WORKER.

During four days of last week the National Council of Women sat in conference at Bristol. This Council is composed of representatives of societies working for social betterment and of its local branches with some elected individual workers, and is federated with twenty-eight other national councils into that International Council which recently met at Christiania. This year its time was largely given to the discussion of resolutions framed and already discussed by some one or more of these societies and branches. When passed, perhaps with amendments, such resolutions are sent round in collected form in order that action may be taken with regard to them. This year they chiefly took the form of amendments of the law which the considered opinion of women will require to be made. No doubt over-much importance may be attached to legislation as a means of social reform, but many measures needed and affecting the lives especially of women and children are long overdue. The College of Nursing, it may be added, has three representatives on the Council.

Naturally the Council desires to see women better represented in Parliament. The experience of the home-maker and of the person most responsible for infant life needs a direct voice in the counsels of the nation not less than any other interest in a day of much domestic legislation. One resolution seeks to gain votes for women on the terms under which men have them, and the Council's branches are to make efforts to discover and assist suitable women candidates, but on the conditions that they shall run no candidate themselves, shall adopt no party bias, and shall not promote the standing of a woman merely *quâ* woman in rivalry with a man. They urge the appointment of more women on Leagues of Nations bodies, and ask that Government shall put into force promptly the Orders in Council already made for the appointment of policewomen, whose preventive work especially has been amply tested. They press also for the enforcement of local by-laws regarding child-labour. Serious detriment to health and education is being threatened in places where evasion goes unheeded.

Amendment of the law dealing with illegitimate children is asked, the process of affiliation to be facilitated and reciprocity with the Dominions secured to prevent evasion of the father by emigration. Ireland has for the present disfranchised herself, but her urgent need is a law on this point. Actually a man can still only be sued on the ground of loss of services to an employer or parent. The legalisation of adoption is also sought, a request which may strengthen the hands of the Departmental Committee now sitting to consider this question. Some period of probation, however, seems to be desirable to protect the child from exploitation or unhappiness before the adoption is finally concluded. The Guardianship of Infants Bill, placing mother in the same position as father, should also be pressed forward.

A difficult question was raised by the resolution desiring that unsworn and uncorroborated evidence of very young children should be accepted—happily, *at the discretion of the magistrate*—in cases of criminal assault. Romancing by children and blackmail by parents are not at all unknown things; on the other hand, in one quoted case, conviction was not secured till a seventh assault, when a life-sentence was inflicted. Lord Lytton took up at a public meeting the resolution desiring widows' pensions on Judge Neil's plan. One voice cried in the wilderness for a contributory scheme on sound life-insurance principles in days of high wages; but the suggestion was not embodied in an amendment. A resolution dealing with the urgent needs of voluntary hospitals was, however, amended to add to the demand for State aid, the addition of paying wards and payments by patients. A much-needed legal amendment directly concerned with health asks for the establishment of a uniform medical standard for emigrants. Very hard cases have occurred in consequence of some accepted persons being rejected on arrival, and left stranded, or returned with homes lost and resources exhausted. Other resolutions dealt with women's Civil Service grievances, the unfairness of taxing together the incomes of married couples, proportional representation, and the State regulation of vice.

Lady Barrett addressed a public meeting on health problems of the moment, and continuation schools and recreation in their connection with health were also then discussed. Lord Buckmaster's Divorce Bill was the subject of another meeting, but no resolution was proposed, and the most salient feature of the discussion was evident ignorance both of the present position and of the provisions and probable results of the proposed Act. As usual, also, the claims of children practically orphaned by divorces were little touched upon. The general sense of the meeting, however, did not appear friendly to wider facilities.

Bristol is noteworthy for its advances in health work, infant care, and education, and the delegates had valuable opportunities of learning, as well as a most hospitable welcome from residents and the public authorities, and delightful excursions in the beautiful surrounding country.

